# Lack of Association between Postoperative Pancreatitis and Other Postoperative Complications Following Pancreaticoduodenectomy

**DOI:** 10.3390/jcm10061179

**Published:** 2021-03-11

**Authors:** Daegwang Yoo, Seo Young Park, Dae Wook Hwang, Jae Hoon Lee, Ki Byung Song, Woohyung Lee, Yejong Park, Eunsung Jun, Song Cheol Kim

**Affiliations:** 1 Division of Hepatobiliary and Pancreatic Surgery, Department of Surgery, Asan Medical Center, University of Ulsan College of Medicine, Seoul 05505, Korea; yoodaegwang@naver.com (D.Y.); gooddr23@naver.com (J.H.L.); mtsong21c@naver.com (K.B.S.); ywhnet@gmail.com (W.L.); blackpig856@gmail.com (Y.P.); eunsungjun@amc.seoul.kr (E.J.); drksc@amc.seoul.kr (S.C.K.); 2 Department of Clinical Epidemiology and Biostatistics, Asan Medical Center, University of Ulsan College of Medicine, Seoul 05505, Korea; biostat81@gmail.com

**Keywords:** pancreatitis, pancreaticoduodenectomy, pancreatic fistula, postoperative complications, amylases

## Abstract

Background: Prediction of post-pancreaticoduodenectomy (PD) morbidity is difficult, especially in the early postoperative period when CT (Computed Tomography) scans are not available. Elevated serum amylase and lipase in postoperative day 0 or 1 may be used to define postoperative acute pancreatitis (POAP), but the existing literature does not agree on whether POAP is significantly associated with postoperative pancreatic fistula (POPF). Methods: We analyzed the data obtained from a previously published randomized controlled trial. POAP was defined as elevations in serum amylase above 110 U/L on postoperative day 0 or 1. Clinically relevant POAP (CR-POAP) was defined as elevations in C-reactive protein level (CRP) on postoperative day 2 in those with POAP. Postoperative complications including severe complications (Clavien–Dindo ≥ IIIa), POPF, and clinically relevant POPF (CR-POPF) were analyzed. Results: In 246 patients, POAP did not show significant associations with total postoperative complications (odds ratio (OR) 0.697; 95% CI, 0.360–1.313; *p* = 0.271), severe complications (OR 0.647; 95% CI, 0.258–1.747; *p* = 0.367), and CR-POPF (OR 0.998; 95% CI, 0.310–3.886; *p* = 0.998) in multivariable analysis. Conclusions: In patients undergoing PD, POAP was not significantly associated with postoperative complications including POPF. Caution should be taken when using POAP as a predictor of POPF.

## 1. Introduction

Pancreaticoduodenectomy (PD) is the mainstay curative treatment for a variety of periampullary diseases [1] but has a rather high postoperative complication rate because of the soft and friable texture of the pancreas and complexity of the procedure. Moreover, it is very difficult to predict the morbidity after PD, especially in the early period such as postoperative day 0 or 1 when postoperative CT (Computed Tomography) scans are not available.

Connor [2] proposed defining postoperative pancreatitis as the elevation of urinary trypsinogen-2 (UTRP-2) over 50 ug/L at postoperative days 1 and 2, or when UTRP-2 is not available, elevations of serum amylase and lipase over the upper limit of normal range at postoperative days 0 and 1. Using the latter definition, Bannone et al. defined the postoperative acute pancreatitis (POAP) and applied it to their retrospective cohort to suggest that POAP is associated with postoperative pancreatic fistula (POPF) and postoperative morbidity [3]. Several other retrospective studies made similar observations on the impact of POAP on postoperative complications following PD [4,5,6,7]. In contrast, a randomized clinical trial showed that elevated serum amylase (>140 U/L) on postoperative day 1 was not significantly associated with POPF [8]. In the present study, we reviewed the data from a previously published single-institution randomized controlled trial [9] to evaluate the association between POAP and postoperative complications including clinically relevant POPF (CR-POPF).

## 2. Methods

### 2.1. Patient Database

This study is a secondary analysis of the data collected in a single-institution randomized controlled trial on the effect of an enhanced recovery after surgery (ERAS) program on patients undergoing PD [9]. The details of the trial design and methods have been previously published [9]. Among the data collected, the following details on patient demographics, surgical variables, and postoperative outcomes were analyzed: age at operation, sex, body mass index (BMI), American Society of Anesthesiologists (ASA) score [10], preoperative laboratory data including tumor markers (carcinoembryonic antigen (CEA) and carbohydrate antigen 19-9 (CA 19-9)), preoperative biliary or pancreatic drainage, preoperative cholangitis, pancreatic duct size, pancreatic texture, operative time, pancreaticojejunostomy (PJ) method (duct-to-mucosa or dunking), tumor type (adenocarcinoma or others), tumor location (ampulla of Vater, distal bile duct, duodenum, pancreas), resection margin status, serum amylase level on postoperative days 0 and 1, and C-reactive protein level (CRP) level on postoperative day 2. As for postoperative complications, the total numbers of any postoperative complications, POPF, delayed gastric emptying, and post-pancreatectomy hemorrhage were analyzed.

The original trial was registered in ClinicalTrials.gov (NCT02372331) and obtained approval from the institutional review board of Asan Medical Center (#2014-0961), which exempted the need for approval for the use of de-identified data in this current analysis (#2019-0210).

### 2.2. Definitions

POAP was defined according to the criteria proposed by Connor [2]. Specifically, any postoperative elevation in serum amylase above the upper limit of normal (110 U/L) on postoperative days 0 or 1 was defined as POAP. Clinically relevant POAP (CR-POAP) was defined as the elevation of CRP (>180 mg) on postoperative day 2 in those with POAP [2,11].

Postoperative complications were classified using the Clavien–Dindo classification system [12], and severe postoperative complications were defined as those equal to or higher than grade IIIa or those that required interventions. POPF was defined and graded according to the recommendations of the International Study Group on Pancreatic Fistula [13], and CR-POPF was defined as grade B or C POPF. All morbidities had been judged by two independent surgeons as described in the published study [9].

### 2.3. Statistical Analysis

Continuous variables were compared using the Student’s t-test and presented as means and standard deviations. Categorical variables were analyzed using the χ2 test and presented as counts and percentages. Univariable and multivariable logistic regression analyses were performed to investigate the effect of POAP and other covariates on each binary outcome. To select the variables in the final multivariable model in a robust and objective manner, we drew 500 bootstrap samples from the original data and performed backward elimination while forcing POAP to be included in the model on each bootstrap sample; the covariates that remained in the model after backward elimination on more than 40% of the bootstrap samples were selected for the final model [14,15]. We carried out this process separately for each outcome. We also used backward elimination to select the variables in an objective, reproducible, and consistent way for all outcomes. In addition, the repeated backward elimination on 500 bootstrap samples was used to obtain a robust result that is not affected by small perturbation of the data. Odds ratio point estimates and 95% confidence intervals (CIs) are presented and differences with *p* values ≤ 0.05 were considered statistically significant. All statistical analyses were conducted using IBM SPSS Statistics for Windows, version 21.0 (IBM Corp., Armonk, NY, USA) and R 3.5.1 (R Foundation for Statistical Computing, Vienna, Austria).

## 3. Results

### 3.1. Patient Demographics

**Figure 1:**
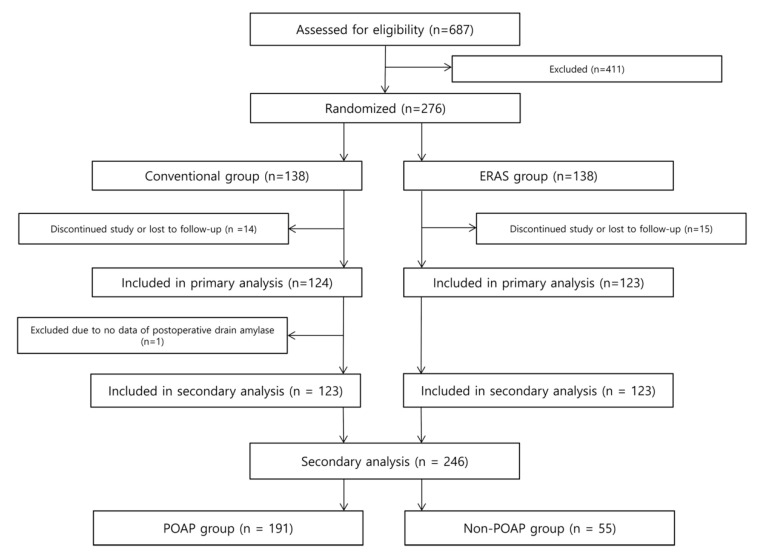
Flow diagram showing the inclusion and exclusion criteria of original and secondary analysis.

A total of 276 patients were enrolled and equally allocated to the conventional group and the intervention group in the original trial [9]. In this initial trial, adult (≥18 and <80 years of age) patients with periampullary cancer or benign lesion were enrolled [9]. Participants were eligible for enrollment if they had to undergo open PD for cure because of resectable periampullary lesion and they had well-preserved bone marrow function, liver function, and renal function (WBC (White blood cell) at least 3000/mm^3^ or absolute neutrophil count at least 1500/mm^3^, platelet count at least 125,000/mm^3^, AST (Aspartate Aminotransferase) and ALT (Alanine Aminotransferase) less than 3 times upper limit of normal, serum creatinine no greater than 1.5 times upper limit of normal) [9]. Exclusion criteria were distant metastasis, recurred periampullary cancer, active or uncontrolled infectious disease, severe psychological or neurological disease, alcohol or drug addiction, overlapping with other clinical trials, pregnancy, uncontrolled cardiopulmonary disease, comorbidities that could affect the quality of life and nutritional status (e.g., liver cirrhosis, renal failure), previous history of major abdominal surgery (e.g., gastric resection or colonic resection), need for simultaneous adjacent organ resection (e.g., portal vein, superior mesenteric vein, transverse colon, and liver) and plan to perform minimally invasive PD. Among 411 patients, 389 were excluded because they met an exclusion criterion, 16 declined to participate, and six were excluded due to other reasons [9]. After applying the exclusion criteria, 246 patients were analyzed (Figure 1), of whom 94 (38.2%) were female and 152 (61.8%) were male, and 191 (77.6%) had POAP after PD. Among 246 patients, 179 had ductal adenocarcinomas. The remaining 67 had intraductal papillary mucinous neoplasm (13.8%, 34/246), neuroendocrine tumors (4.5%, 11/246), gastrointestinal tumors (3.3%, 8/246), serous cystadenomas (1.2%, 3/246), chronic pancreatitis or cholangitis (1.2%, 3/246), tubular adenoma (0.8%, 2/246), pancreatic intraepithelial neoplasia (0.8%, 2/246), mucinous cystic neoplasm (0.4%, 1/246), solid pseudopapillary neoplasm (0.4%, 1/246), acinar cell carcinoma (0.4%, 1/246), and pancreatolithiasis (0.4%, 1/246).

### 3.2. Comparative Analysis of Basic Characteristics and Postoperative Complications

The demographic characteristics and preoperative factors of the patients according to the presence of POAP are listed in Table 1. The two groups did not show significant differences in terms of age at operation, sex, BMI, ASA score, preoperative CEA and CA 19-9, preoperative biliary or pancreatic drainage, preoperative cholangitis, ERAS group, PJ method, tumor type, cancer or non-cancer, and resection margin status. However, there were significant differences in the distribution of tumor location (*p* = 0.004) between the two groups, and the POAP group had smaller pancreatic duct size (*p* = 0.002) and were more likely to have soft pancreatic texture (*p* < 0.001) (Table 1).

Total complication rates were 54.5% (134/246). Among these, grade I complications (Clavien–Dindo classification) accounted for 40.3% (52/134), grade II complications accounted for 44.8% (60/134), grade III complications accounted for 11.1% (15/134), and grade IV complications accounted for 3.7% (5/134). Table 2 shows the postoperative complication rates of the two groups. There were no significant differences in terms of the rate of total postoperative complications (*p* = 0.257), severe complications (Clavien–Dindo ≥ IIIa) (*p* = 0.333), CR-POPF (*p* = 1.000), delayed gastric emptying (*p* = 0.685), and post-pancreatectomy hemorrhage (*p* = 1.000) (Table 2). Beyond CR-POPF, delayed gastric emptying, and post-pancreatectomy hemorrhage, there were other complications in severe complications (Clavien–Dindo classification ≥ IIIa), such as intraabdominal fluid collection (3.8%, 5/131), pneumonia (1.5%, 2/131), afferent loop ischemia (1.5%, 2/131), myocardiac infarction (0.8%, 1/131), surgical site infection which needed to be repaired under general anesthesia (0.8%, 1/131), choledochojejunostomy site stenosis (0.8%, 1/131), and superior mesenteric vein stenosis (0.8%, 1/131). These complications were not significantly different between the two groups.

### 3.3. Association between POAP and Postoperative Complications

Table 3 shows the results of univariable and multivariable analyses on total postoperative complication rate after backward elimination by forcing POAP. POAP was not significantly associated with total postoperative complication rate in both univariable analysis (odds ratio (OR) 0.703; 95% CI, 0.378–1.286; *p* = 0.256) and multivariable analysis (OR 0.735; 95% CI, 0.393–1.356; *p* = 0.327).

POAP also did not show significant associations with severe complications in univariable analysis (OR 0.574; 95% CI, 0.239–1.481; *p* = 0.227) and multivariable analysis (OR 0.647; 95% CI, 0.258–1.747; *p* = 0.367) (Table 4). Lastly, POAP was not a significant predictor of CR-POPF in univariable analysis (OR 1.008; 95% CI, 0.344–3.677; *p* = 0.989) and multivariable analysis (OR 0.998; 95% CI, 0.310–3.886, *p* = 0.998) (Table 5).

### 3.4. Association between CR-POAP and Postoperative Complications

We also performed univariable and multivariable analyses of postoperative complications after backward elimination by forcing CR-POAP. Similar to aforementioned results, CR-POAP was not significantly associated with total postoperative complication rate in univariable analysis (OR 0.888; 95% CI, 0.520–1.516; *p* = 0.662) and multivariable analysis (OR 0.897; 95% CI, 0.517–1.555; *p* = 0.697) (Supplementary Table S1).

CR-POAP did not show significant associations with severe complications in univariable analysis (OR 0.788; 95% CI, 0.295–1.899; *p* = 0.611) and multivariable analysis (OR 0.854; 95% CI, 0.306–2.182; *p* = 0.750) (Supplementary Table S2). Lastly, CR-POAP was not a significant predictor of CR-POPF in univariable analysis (OR 0.785; 95% CI, 0.244–2.165; *p* = 0.656) and multivariable analysis (OR 0.758; 95% CI, 0.216–2.364, *p* = 0.644) (Supplementary Table S3).

We also conducted a sensitivity analysis after excluding three patients with high preoperative amylase levels (>110 U/L). No significant associations were found between CR-POAP and total postoperative complication rate (Supplementary Table S4), severe postoperative complications (Supplementary Table S5), and CR-POPF (Supplementary Table S6) in univariable and multivariable analyses.

## 4. Discussion

In this secondary data analysis from a single-institution randomized controlled trial, POAP and CR-POAP did not show any significant association with the rate of total postoperative complication, severe complication, or CR-POPF. This result remained unchanged after excluding patients with high preoperative amylase levels.

CR-POAP is defined by both serum lipase levels on postoperative days 0 or 1 and serum CRP [2], and several studies claimed that CR-POAP is significantly associated with POPF [6,11,16,17]. However, these studies had variations in the timing of postoperative serum CRP measurement, and a meta-analysis concluded that CRP only has limited value in predicting POPF [18]. In line with the meta-analysis, our study showed that CR-POAP did not show any significant associations with total postoperative complications, severe postoperative complications, and CR-POPF in both univariable and multivariable analyses, even after excluding patients with high preoperative serum amylase levels.

Elevation in serum amylase level could be due to insult to the pancreas, which may range from simple manipulation of the pancreatic duct to severe pancreatitis [19]. Moreover, serum amylase can be elevated due to a wide variety of disorders including postoperative lactic acidosis, acute kidney injury, and primary diseases such as chronic alcoholism, anorexia nervosa, bulimia, and salivary gland disease [19]. Therefore, clinicians should not jump to the conclusion that hyperamylasemia is directly connected to POPF. This holds true even after adding serum CRP elevation to the definition of POAP, because CRP is an acute-phase reactant protein that can be elevated due to any kind of inflammatory or infectious process [20,21].

Male sex is one of the most important risk factors of postoperative complications [22]. Multivariable analyses of severe complications (Clavien–Dindo ≥ IIIa) after backward elimination by forcing the inclusion of POAP showed female sex significantly lowered the risk of severe complications (Table 4). The same analysis between CR-POAP and postoperative severe complications (Clavien–Dindo ≥ IIIa) showed that female sex lowered the risk of severe complications (Supplementary Table S2). Finally, multivariable analyses of CR-POPF after backward elimination by forcing the inclusion of POAP showed that male sex can be a significant risk factor of CR-POPF (Table 5), which is consistent with previous studies [3,5]. On the other hand, some factors did not show any significant association with postoperative complications or POPF, including BMI and ASA, which were significant risk factors of serious complications or POPF in previous studies [3,22]. However, BMI > 25 kg/m^2^ was used as a variable in previous studies [3,5,22], but the mean and the standard of BMI in Asian patients of our study may be different from the previous studies’. ASA was also a significant risk factor in one article [22], but it was not in another [3]. Furthermore, age was not a significant risk factor of total or severe complications (Table 3 and Table 4) but was a significant risk factor of CR-POPF (Table 5) in our study. Because of these inconsistent results, randomized controlled trials are needed in the future.

This study has limitations inherent to its single-institution, retrospective design; nevertheless, unlike most retrospective cohort studies, this study utilized the data derived from a randomized controlled trial, which provides a better quality of data by reducing the risk of measurement bias and selection bias [23,24,25,26,27]. We also used bootstrap samples and performed backward elimination while forcing POAP to be included in the model on each bootstrap sample. By doing this, we can select the variables in an objective, reproducible, and consistent way for all outcomes. Furthermore, the repeated backward elimination on 500 bootstrap samples was used to obtain a robust result that is not affected by small perturbation of the data. Moreover, the complication rates such as CR-POPF after PD were rather low, so it may be possible that the event rate was not enough to delineate a significant relationship between POAP and complications. A possible reason for the low complication rate is the proficiency of surgeons at our institution; surgeons who participated in the original randomized clinical trial were highly experienced, as they had collectively carried out more than 200 cases of PD during the 5 years before the start of the study [9]. Therefore, our data can be regarded as well-representing the tertiary medical center’s setting without the confounding effect of relatively unexperienced surgeons under a learning curve. Lastly, the majority (73%) of our study patients were a high-risk group according to the fistula risk score system [28,29], whereas high-risk patients only comprised 38% of the study population in another recent study [3]. Our study may be more appropriate in elucidating the association between POAP and POPF because it had a higher proportion of high-risk patients. Nevertheless, a large-sized, randomized, controlled trial specifically designed to test the association between POAP and POPF would be invaluable in drawing a more firm conclusion on this matter.

In conclusion, POAP was not significantly associated with postoperative complications including CR-POPF in patients undergoing PD. Therefore, although POAP may be easily diagnosed by measuring serum amylase and serum CRP, caution should be taken when using POAP as a predictor of POPF.

## Figures and Tables

**Table 1:** Demographic characteristics and preoperative factors.

	POAP	Non-POAP	*p* Value
	(*n* = 191)	(*n* = 55)	
Age, years (mean ± SD)	62.8 ± 9.4	63.9 ± 8.3	0.461
Sex, *n* (%)			0.268
Female	77 (40.3)	17 (30.9)	
Male	114 (59.7)	38 (69.1)	
BMI, kg/m^2^ (mean ± SD)	24.4 ± 3.1	24.0 ± 3.0	0.404
ASA score, *n* (%)			0.448
Grade I	21 (11.0)	6 (10.9)	
Grade II	156 (81.7)	42 (76.4)	
Grade ≥ III	14 (7.3)	7 (12.7)	
Preoperative CEA (mean ± SD)	168.4 ± 221.6	177.8 ± 296.2	0.798
Preoperative CA 19-9 (mean ± SD)	0.77 ± 0.18	0.78 ± 0.21	0.801
Preoperative biliary/pancreatic drainage, *n* (%)			0.409
No	83 (43.5)	28 (50.9)	
Yes	108 (56.5)	27 (49.1)	
Preoperative cholangitis, *n* (%)			0.733
No	150 (78.5)	45 (81.8)	
Yes	41 (21.5)	10 (18.2)	
ERAS group, *n* (%)			1.000
Conventional group	95 (49.7)	28 (50.9)	
Intervention group	96 (50.3)	27 (49.1)	
Preoperative pancreatic duct size (mean ± SD)	3.2 ± 2.1	4.1 ± 2.1	0.005
Pancreatic texture			< 0.0001
Soft	138 (74.2)	23 (41.8)	
Firm	48 (25.8)	32 (58.2)	
Pancreaticojejunostomy method, *n* (%)			1.000
Duct-to-mucosa	172 (90.1)	49 (89.1)	
Dunking	19 (9.9)	6 (10.9)	
Tumor type, *n* (%)			0.869
Adenocarcinoma	138 (72.3)	41 (74.5)	
Others	53 (27.7)	14 (25.5)	
Tumor location, *n* (%)			0.004
Ampulla of Vater	32 (16.8)	8 (14.5)	
Distal bile duct	61 (31.9)	6 (10.9)	
Duodenum	12 (6.3)	2 (3.6)	
Pancreas	86 (45.0)	39 (70.9)	
Cancer, *n* (%)			1.000
No	48 (25.1)	14 (25.5)	
Yes	143 (74.9)	41 (74.5)	
Retrieved lymph nodes in adenocarcinoma patients (mean ± SD)	20.4 ± 8.6	19.1 ± 7.6	0.371
Resection margin status, *n* (%)			0.948
R0	174 (91.0)	51 (92.5)	
R1	17 (9.0)	4 (7.5)	

**Table 2:** Postoperative complications.

	POAP	Non-POAP	*p* Value
	(*n* = 191)	(*n* = 55)	
Total complications			0.255
No	93 (48.7)	22 (40.0)	
Yes	98 (51.3)	33 (60.0)	
Severe complications (Clavien–Dindo ≥ IIIa)			0.333
No	174 (91.1)	47 (85.5)	
Yes	17 (8.9)	8 (14.5)	
CR-POPF (Grade B or C)			1.000
No	177 (92.7)	51 (92.7)	
Yes	14 (7.3)	4 (7.3)	
Delayed gastric emptying			0.685
No	182 (95.3)	51 (92.7)	
Yes	9 (4.7)	4 (7.3)	
Post-pancreatectomy hemorrhage			1.000
No	187 (97.9)	54 (98.2)	
Yes	4 (2.1)	1 (1.8)	

**Table 3:** Univariable and multivariable analyses of total postoperative complication rate after backward elimination by forcing the inclusion of postoperative acute pancreatitis (POAP).

		Univariable		Multivariable	
Variable		Odds Ratio (95% Confidence Interval)	*p* Value	Odds Ratio (95% Confidence Interval)	*p* Value
POAP		0.703 (0.378–1.286)	0.256	0.735 (0.393–1.356)	0.327
Age		1.015 (0.988–1.044)	0.280		
Sex (Ref: Male)		0.867 (0.518–1.453)	0.589		
BMI		0.954 (0.877–1.036)	0.264		
ASA score (Ref: I)			0.151		0.163
	II	1.383 (0.617–3.159)	0.432	1.421 (0.630–3.268)	0.399
	III	3.125 (0.959–11.130)	0.066	3.110 (0.946–11.158)	0.069
Preoperative CEA		1.001 (1.000–1.002)	0.163		
Preoperative CA19-9		0.999 (0.262–3.820)	0.998		
Preoperative biliary/pancreatic drainage		0.883 (0.532–1.460)	0.627	0.870 (0.520–1.452)	0.595
Preoperative cholangitis		0.891 (0.480–1.657)	0.715		
ERAS group		0.907 (0.549–1.497)	0.701		
Preoperative pancreatic duct size		0.982 (0.866–1.112)	0.771		
Pancreatic texture		0.806 (0.468–1.382)	0.435		
Pancreaticojejunostomy method		1.358 (0.591–3.246)	0.477		
Tumor location (Ref: Pancreas)			0.718		
	Ampulla of Vater	0.686 (0.332–1.400)	0.302		
	Distal common bile duct	1.034 (0.570–1.884)	0.913		
	Duodenum	1.118 (0.367–3.572)	0.845		
Cancer		1.001 (0.560–17.783)	0.996		
Resection margin status		1.484 (0.601–3.881)	0.400		

**Table 4:** Univariable and multivariable analyses of severe complications (Clavien–Dindo ≥ IIIa) after backward elimination by forcing the inclusion of POAP.

		Univariable		Multivariable	
Variable		Odds Ratio (95% Confidence Interval)	*p* Value	Odds Ratio (95% Confidence Interval)	*p* Value
POAP		0.574 (0.239–1.481)	0.227	0.647 (0.258–1.747)	0.367
Age		1.034 (0.987–1.089)	0.178	1.046 (0.993–1.109)	0.108
Sex (Ref: Male)		0.477 (0.168–1.179)	0.130	0.316 (0.099–0.903)	0.038
BMI		1.003 (0.872–1.146)	0.971		
ASA score (Ref: I)			0.044		
	II	NA *	0.989		
	III	NA *	0.990		
Preoperative CEA		1.000 (0.998–1.002)	0.866		
Preoperative CA19-9		0.661 (0.066–5.778)	0.717	0.157 (0.011–1.905)	0.154
Preoperative biliary/pancreatic drainage		0.735 (0.317–1.692)	0.467	0.535 (0.212–1.314)	0.175
Preoperative cholangitis		0.705 (0.199–1.962)	0.540		
ERAS group		1.569 (0.683–3.752)	0.294		
Preoperative pancreatic duct size		1.018 (0.805–1.208)	0.856		
Pancreaticojejunostomy method		1.233 (0.277–3.940)	0.749		
Pancreatic texture		0.811 (0.343–2.013)	0.637		
Tumor location (Ref: Pancreas)			0.570		
	Ampulla of Vater	0.386 (0.059–1.453)	0.220		
	Distal common bile duct	0.856 (0.312–2.148)	0.748		
	Duodenum	0.564 (0.030–3.160)	0.594		
Cancer		1.390 (0.534–4.330)	0.529		
Resection margin status		3.125 (0.945–8.976)	0.043	3.451 (0.987–10.794)	0.039

* The results could not be calculated due to the existence of zero cells.

**Table 5:** Univariable and multivariable analyses of clinically relevant postoperative pancreatic fistula (CR-POPF) after backward elimination by forcing the inclusion of POAP.

		Univariable		Multivariable	
Variable		Odds Ratio (95% Confidence Interval)	*p* Value	Odds Ratio (95% Confidence Interval)	*p* Value
POAP		1.008 (0.344–3.677)	0.989	0.998 (0.310–3.886)	0.998
Age		1.064 (1.003–1.137)	0.051	1.075 (1.008–1.154)	0.036
Sex (Ref: Male)		0.301 (0.068–0.944)	0.063	0.263 (0.056–0.888)	0.050
BMI		1.017 (0.866–1.186)	0.838		
ASA score (Ref: I)			0.110		
	II	NA *	0.990		
	III	NA *	0.989		
Preoperative CEA		1.002 (1.000–1.003)	0.016		
Preoperative CA19-9		4.822 (0.414–48.984)	0.192		
Preoperative biliary/pancreatic drainage		3.095 (1.073–11.175)	0.052		
Preoperative cholangitis		0.750 (0.169–2.388)	0.660	0.384 (0.078–1.363)	0.177
ERAS group		0.359 (0.112–0.985)	0.059	0.347 (0.102–1.017)	0.066
Preoperative pancreatic duct size		0.832 (0.555–1.099)	0.298		
Pancreaticojejunostomy method		1.114 (0.169–4.260)	0.890		
Pancreatic texture		0.993 (0.370–2.949)	0.990		
Tumor location (Ref: Pancreas)			0.011		
	Ampulla of Vater	1.263 (0.176–6.125)	0.785		
	Distal common bile duct	4.714 (1.633–15.56)	0.006		
	Duodenum	NA *	0.989		
Cancer		1.746 (0.552–7.726)	0.392		
Resection margin status		3.462 (0.906–10.956)	0.045	2.970 (0.700–11.004)	0.113

* The results could not be calculated due to the existence of zero cells.

## Supplementary Materials

The following are available online at https://www.mdpi.com/2077-0383/10/6/1179/s1, Supplementary Table S1: Univariable and multivariable analyses of total postoperative complication rate after backward elimination by forcing the inclusion of clinically relevant POAP, Supplementary Table S2: Univariable and multivariable analyses of severe complications (Clavien–Dindo ≥ IIIa) rate after backward elimination by forcing the inclusion of clinically relevant POAP, Supplementary Table S3: Univariable and multivariable analyses of CR-POPF rate after backward elimination by forcing the inclusion of clinically relevant POAP, Supplementary Table S4: Univariable and multivariable analyses of total postoperative complication rate after backward elimination by forcing the inclusion of clinically relevant POAP after excluding patients with high preoperative amylase levels, Supplementary Table S5: Univariable and multivariable analyses of severe complications (Clavien–Dindo ≥ IIIa) after backward elimination by forcing the inclusion of clinically relevant POAP after excluding patients with high preoperative amylase levels, Supplementary Table S6: Univariable and multivariable analyses of CR-POPF after backward elimination by forcing the inclusion of clinically relevant POAP after excluding patients with high preoperative amylase levels.

## References

[1] Whipple A.O. (1946). Observations on radical surgery for lesions of the pancreas. Surg. Gynecol. Obstet..

[2] Connor S. (2016). Defining post-operative pancreatitis as a new pancreatic specific complication following pancreatic resection. HPB.

[3] Bannone E., Andrianello S., Marchegiani G., Masini G., Malleo G., Bassi C., Salvia R. (2018). Postoperative acute pancreatitis following pancreaticoduodenectomy: A determinant of fistula potentially driven by the intraoperative fluid management. Ann. Surg..

[4] Kühlbrey C.M., Samiei N., Sick O., Makowiec F., Hopt U.T., Wittel U.A. (2017). Pancreatitis after pancreatoduodenectomy predicts clinically relevant postoperative pancreatic fistula. J. Gastrointest. Surg..

[5] Palani Velu L.K., Chandrabalan V.V., Jabbar S., McMillan D.C., McKay C.J., Carter C.R., Jamieson N.B., Dickson E.J. (2014). Serum amylase on the night of surgery predicts clinically significant pancreatic fistula after pancreaticoduodenectomy. HPB.

[6] Palani Velu L.K., McKay C.J., Carter C.R., McMillan D.C., Jamieson N.B., Dickson E.J. (2016). Serum amylase and C-reactive protein in risk stratification of pancreas-specific complications after pancreaticoduodenectomy. Br. J. Surg..

[7] Cloyd J.M., Kastenberg Z.J., Visser B.C., Poultsides G.A., Norton J.A. (2014). Postoperative serum amylase predicts pancreatic fistula formation following pancreaticoduodenectomy. J. Gastrointest. Surg..

[8] Jin S., Shi X.J., Wang S.Y., Zhang P., Lv G.Y., Du X.H., Wang G.Y. (2017). Drainage fluid and serum amylase levels accurately predict development of postoperative pancreatic fistula. World J. Gastroenterol..

[9] Hwang D.W., Kim H.J., Lee J.H., Song K.B., Kim M.H., Lee S.K., Choi K.T., Jun I.G., Bang J.Y., Kim S.C. (2019). Effect of enhanced recovery after surgery program on pancreaticoduodenectomy: A randomized controlled trial. J. Hepatobiliary Pancreat. Sci..

[10] Owens W.D., Felts J.A., Spitznagel E.L. (1978). ASA physical status classifications: A study of consistency of ratings. Anesthesiology.

[11] Birgin E., Reeg A., Téoule P., Rahbari N.N., Post S., Reissfelder C., Rückert F. (2019). Early postoperative pancreatitis following pancreaticoduodenectomy: What is clinically relevant postoperative pancreatitis?. HPB.

[12] Dindo D., Clavien P.A. (2008). What is a surgical complication?. World J. Surg..

[13] Bassi C., Marchegiani G., Dervenis C., Sarr M., Abu Hilal M., Adham M., Allen P., Andersson R., Asbun H.J., Besselink M.G. (2017). The 2016 update of the International Study Group (ISGPS) definition and grading of postoperative pancreatic fistula: 11 years after. Surgery.

[14] Harrell F.E. (2001). Regression Modeling Strategies: With Applications to Linear Models, Logistic Regression, and Survival Analysis.

[15] Sauerbrei W., Schumacher M. (1992). A bootstrap resampling procedure for model building: Application to the Cox regression model. Stat. Med..

[16] Partelli S., Pecorelli N., Muffatti F., Belfiori G., Crippa S., Piazzai F., Castoldi R., Marmorale C., Balzano G., Falconi M. (2017). Early postoperative prediction of clinically relevant pancreatic fistula after pancreaticoduodenectomy: Usefulness of C-reactive protein. HPB.

[17] Welsch T., Frommhold K., Hinz U., Weigand M.A., Kleeff J., Friess H., Büchler M.W., Schmidt J. (2008). Persisting elevation of C-reactive protein after pancreatic resections can indicate developing inflammatory complications. Surgery.

[18] Solaini L., Atmaja B.T., Watt J., Arumugam P., Hutchins R.R., Abraham A.T., Bhattacharya S., Kocher H.M. (2015). Limited utility of inflammatory markers in the early detection of postoperative inflammatory complications after pancreatic resection: Cohort study and meta-analyses. Int. J. Surg..

[19] Pieper-Bigelow C., Strocchi A., Levitt M.D. (1990). Where does serum amylase come from and where does it go?. Gastroenterol. Clin. N. Am..

[20] Ballantyne C.M., Nambi V. (2005). Markers of inflammation and their clinical significance. Atheroscler. Suppl..

[21] Nehring S.M., Goyal A., Bansal P., Patel B.C. (2020). C Reactive Protein. StatPearls.

[22] Aoki S., Miyata H., Konno H., Gotoh M., Motoi F., Kumamaru H., Wakabayashi G., Kakeji Y., Mori M., Seto Y. (2017). Risk factors of serious postoperative complications after pancreaticoduodenectomy and risk calculators for predicting postoperative complications: A nationwide study of 17,564 patients in Japan. J. Hepatobiliary Pancreat. Sci..

[23] Cheng H.G., Phillips M.R. (2014). Secondary analysis of existing data: Opportunities and implementation. Shanghai Arch. Psychiatry.

[24] Cole A.P., Trinh Q.D. (2017). Secondary data analysis: Techniques for comparing interventions and their limitations. Curr. Opin. Urol..

[25] Furberg C.D., Friedman L.M. (2012). Approaches to data analyses of clinical trials. Prog. Cardiovasc. Dis..

[26] Castle J.E. (2003). Maximizing research opportunities: Secondary data analysis. J. Neurosci. Nurs..

[27] Martin-Sanchez F.J., Aguiar-Pulido V., Lopez-Campos G.H., Peek N., Sacchi L. (2017). Secondary use and analysis of big data collected for patient care. Yearb. Med. Inform..

[28] Callery M.P., Pratt W.B., Kent T.S., Chaikof E.L., Vollmer C.M. (2013). A prospectively validated clinical risk score accurately predicts pancreatic fistula after pancreatoduodenectomy. J. Am. Coll. Surg..

[29] Miller B.C., Christein J.D., Behrman S.W., Drebin J.A., Pratt W.B., Callery M.P., Vollmer C.M. (2014). A multi-institutional external validation of the fistula risk score for pancreatoduodenectomy. J. Gastrointest. Surg..

