# Supplementary material for: Lack of Association between Postoperative Pancreatitis and Other Postoperative Complications Following Pancreaticoduodenectomy
Source: J Clin Med. 2021 Mar 11;10(6):1179. doi: 10.3390/jcm10061179 (PMC8001526; doi:10.3390/jcm10061179)
Supplement: Supplementary file 1 [file jcm-10-01179-s001.pdf]

Supplementary table 1. Univariable and multivariable analyses of total postoperative complication rate after backward elimination by forcing the inclusion of clinically relevant POAP

Variable		Univariable		Multivariable	
		Odds ratio (95% confidence interval)	*P* value	Odds ratio (95% confidence interval)	*P* value
**Clinically relevant POAP**		0.888 (0.520-1.516)	0.662	0.897 (0.517-1.555)	0.697
**Age**		1.015 (0.988-1.044)	0.280		
**Sex**		0.867 (0.518-1.453)	0.589		
**BMI**		0.954 (0.877-1.036)	0.264		
**ASA score (Ref: I)**			0.151		0.141
	**II**	1.383 (0.617-3.159)	0.432	1.440 (0.637-3.318)	0.382
	**III**	3.125 (0.959-11.130)	0.066	3.238 (0.988-11.597)	0.059
**Preoperative CEA**		1.001 (1.000-1.002)	0.163		
**Preoperative CA19-9**		0.999 (0.262-3.820)	0.998		
**Preoperative biliary / pancreatic drainage**		0.883 (0.532-1.460)	0.627	0.871 (0.518-1.462)	0.602
**Preoperative cholangitis**		0.891 (0.480-1.657)	0.715		
**ERAS group**		0.907 (0.549-1.497)	0.701		
**Preoperative pancreatic duct size**		0.982 (0.866-1.112)	0.771		
**Pancreatic texture**		0.806 (0.468-1.382)	0.435		
**Pancreaticojejunostomy method**		1.358 (0.591-3.246)	0.477		
**Tumor location (Ref: Pancreas)**			0.718		
	**Ampulla of Vater**	0.686 (0.332-1.400)	0.302		
	**Distal common bile duct**	1.034 (0.570-1.884)	0.913		
	**Duodenum**	1.118 (0.367-3.572)	0.845		
**Cancer**		1.001 (0.560-1.783)	0.996		
**Resection margin status**		1.484 (0.601-3.881)	0.400		

Supplementary table 2. Univariable and multivariable analyses of severe complications (Clavien-Dindo ≥ IIIa) rate after backward elimination by forcing the inclusion of clinically relevant POAP

Variable		Univariable		Multivariable	
		**Odds ratio (95% confidence interval)**	***P* value**	**Odds ratio (95% confidence interval)**	***P* value**
**Clinically relevant POAP**		0.788 (0.295-1.899)	0.611	0.854 (0.306-2.182)	0.750
**Age**		1.034 (0.987-1.089)	0.178		
**Sex**		0.477 (0.168-1.179)	0.130	0.331 (0.104-0.939)	0.046
**BMI**		1.003 (0.872-1.146)	0.971		
**ASA score (Ref: I)**			0.044		
	**II**	NA*	0.989		
	**III**	NA*	0.990		
**Preoperative CEA**		1.000 (0.998-1.002)	0.866		
**Preoperative CA19-9**		0.661 (0.066-5.778)	0.717	0.205 (0.013-2.757)	0.242
**Preoperative biliary / pancreatic drainage**		0.735 (0.317-1.692)	0.467	0.601 (0.242-1.456)	0.261
**Preoperative cholangitis**		0.705 (0.199-1.962)	0.540		
**ERAS group**		1.569 (0.683-3.752)	0.294		
**Preoperative pancreatic duct size**		1.018 (0.805-1.208)	0.856		
**Pancreaticojejunostomy method**		1.233 (0.277-3.940)	0.749		
**Pancreatic texture**		0.811 (0.343-2.013)	0.637		
**Tumor location (Ref: Pancreas)**			0.570		
	**Ampulla of Vater**	0.385 (0.059-1.453)	0.220		
	**Distal common bile duct**	0.856 (0.312-2.148)	0.748		
	**Duodenum**	0.564 (0.030-3.160)	0.594		
**Cancer**		1.390 (0.534-4.330)	0.529		
**Resection margin status**		3.125 (0.945-8.976)	0.043	3.507 (1.020-10.742)	0.033

* The results could not be calculated due to the existence of zero cells.

Supplementary table 3. Univariable and multivariable analyses of CR-POPF rate after backward elimination by forcing the inclusion of clinically relevant POAP

Variable		Univariable		Multivariable	
		**Odds ratio (95% confidence interval)**	***P* value**	**Odds ratio (95% confidence interval)**	***P* value**
**Clinically relevant POAP**		0.785 (0.244-2.165)	0.656	0.758 (0.216-2.364)	0.644
**Age**		1.064 (1.003-1.137)	0.051	1.072 (1.006-1.152)	0.043
**Sex**		0.301 (0.068-0.944)	0.063	0.268 (0.058-0.905)	0.053
**BMI**		1.017 (0.866-1.186)	0.838		
**ASA score (Ref: I)**			0.110		
	**II**	NA*	0.990		
	**III**	NA*	0.990		
**Preoperative CEA**		1.002 (1.000-1.003)	0.016		
**Preoperative CA19-9**		4.822 (0.414-48.984)	0.192		
**Preoperative biliary / pancreatic drainage**		3.095 (1.073-11.175)	0.052		
**Preoperative cholangitis**		0.750 (0.169-2.388)	0.660	0.384 (0.078-1.367)	0.178
**ERAS group**		0.359 (0.112-0.985)	0.059	0.364 (0.106-1.082)	0.082
**Preoperative pancreatic duct size**		0.832 (0.555-1.099)	0.298		
**Pancreaticojejunostomy method**		1.114 (0.169-4.260)	0.890		
**Pancreatic texture**		0.993 (0.370-2.949)	0.990		
**Tumor location (Ref: Pancreas)**			0.011		
	**Ampulla of Vater**	1.263 (0.176-6.125)	0.785		
	**Distal common bile duct**	4.714 (1.633-15.56)	0.006		
	**Duodenum**	NA*	0.989		
**Cancer**		1.746 (0.552-7.726)	0.392		
**Resection margin status**		3.462 (0.906-10.956)	0.045	2.995 (0.704-11.121)	0.111

* The results could not be calculated due to the existence of zero cells.

Supplementary table 4. Univariable and multivariable analyses of total postoperative complication rate after backward elimination by forcing the inclusion of clinically relevant POAP after excluding patients with high preoperative amylase levels

Variable		Univariable		Multivariable	
		**Odds ratio (95% confidence interval)**	***P* value**	**Odds ratio (95% confidence interval)**	***P* value**
**Clinically relevant POAP**		0.595 (0.504-1.481)	0.595	0.875 (0.502-1.523)	0.653
**Age**		1.016 (0.988-1.044)	0.275		
**Sex**		0.847 (0.504-1.423)	0.531		
**BMI**		0.961 (0.884-1.044)	0.352		
**ASA score (Ref: I)**			0.149		0.138
	**II**	1.371 (0.611-3.134)	0.445	1.434 (0.634-3.308)	0.388
	**III**	3.125 (0.959-11.130)	0.066	3.254 (0.993-11.657)	0.058
**Preoperative CEA**		1.001 (1.000-1.002)	0.200		
**Preoperative CA19-9**		1.015 (0.265-3.897)	0.983		
**Preoperative biliary / pancreatic drainage**		0.871 (0.524-1.444)	0.593	0.863 (0.511-1.451)	0.578
**Preoperative cholangitis**		0.899 (0.483-1.672)	0.735		
**ERAS group**		0.890 (0.537-1.473)	0.650		
**Preoperative pancreatic duct size**		0.982 (0.866-1.112)	0.768		
**Pancreatic texture**		0.818 (0.471-1.411)	0.472		
**Pancreaticojejunostomy method**		1.266 (0.543-3.054)	0.588		
**Tumor location (Ref: Pancreas)**			0.731		
	**Ampulla of Vater**	0.684 (0.331-1.398)	0.299		
	**Distal common bile duct**	1.003 (0.550-1.834)	0.992		
	**Duodenum**	1.114 (0.366-3.565)	0.849		
**Cancer**		0.993 (0.555-1.769)	0.980		
**Resection margin status**		1.579 (0.611-4.383)	0.356		

Supplementary table 5. Univariable and multivariable analyses of severe complications (Clavien-Dindo ≥ IIIa) after backward elimination by forcing the inclusion of clinically relevant POAP after excluding patients with high preoperative amylase levels

Variable		Univariable		Multivariable	
		Odds ratio (95% Confidence interval)	*P* value	Odds ratio (95% Confidence interval)	*P* value
**Clinically relevant POAP**		0.789 (0.295-1.900)	0.612	0.819 (0.295-2.067)	0.684
**Age**		1.034 (0.987-1.089)	0.180	1.036 (0.985-1.096)	0.187
**Sex**		0.475 (0.168-1.175)	0.128	0.419 (0.144-1.058)	0.081
**BMI**		1.002 (0.872-1.146)	0.974		
**ASA score (Ref: I)**			0.042		
	**II**	NA*	0.989		
	**III**	NA*	0.990		
**Preoperative CEA**		1.000 (0.998-1.002)	0.822		
**Preoperative CA19-9**		0.655 (0.066-5.703)	0.710		
**Preoperative biliary / pancreatic drainage**		0.754 (0.325-1.735)	0.504	0.594 (0.238-1.447)	0.253
**Preoperative cholangitis**		0.693 (0.195-1.929)	0.520		
**ERAS group**		1.556 (0.677-3.722)	0.304		
**Preoperative pancreatic duct size**		1.017 (0.805-1.207)	0.862		
**Pancreaticojejunostomy method**		0.776 (0.328-1.930)	0.571		
**Pancreatic texture**		1.279 (0.286-4.104)	0.708		
**Tumor location (Ref: Pancreas)**			0.552		
	**Ampulla of Vater**	0.379 (0.058-1.427)	0.211		
	**Distal common bile duct**	0.854 (0.311-2.147)	0.746		
	**Duodenum**	0.554 (0.029-3.103)	0.582		
**Cancer**		1.416 (0.544-4.411)	0.506		
**Resection margin status**		3.554 (1.062-10.413)	0.026	3.999 (1.144-12.558)	0.021

* The results could not be calculated due to the existence of zero cells.

Supplementary table 6. Univariable and multivariable analyses of CR-POPF after backward elimination by forcing the inclusion of clinically relevant POAP after excluding patients with high preoperative amylase levels

		Univariable		Multivariable	
**Variable**		**Odds ratio (95% confidence interval)**	***P* value**	**Odds ratio (95% confidence interval)**	***P* value**
**Clinically relevant POAP**		0.785 (0.244-2.167)	0.657	0.667 (0.184-2.135)	0.510
**Age**		1.064 (1.003-1.137)	0.051	1.082 (1.010-1.170)	0.035
**Sex**		0.300 (0.068-0.941)	0.063	0.198 (0.040-0.710)	0.023
**BMI**		1.016 (0.865-1.186)	0.839	1.171 (0.961-1.432)	0.116
**ASA score (Ref: I)**			0.108		
	**II**	NA*	0.990		
	**III**	NA*	0.990		
**Preoperative CEA**		1.002 (1.000-1.003)	0.014		
**Preoperative CA19-9**		4.744 (0.409-48.042)	0.195		
**Preoperative biliary / pancreatic drainage**		3.174 (1.099-11.462)	0.047		
**Preoperative cholangitis**		0.738 (0.166-2.349)	0.641	0.317 (0.060-1.191)	0.123
**ERAS group**		0.355 (0.111-0.976)	0.056	0.309 (0.083-0.967)	0.056
**Preoperative pancreatic duct size**		0.833 (0.557-1.099)	0.298		
**Pancreaticojejunostomy method**		0.953 (0.355-2.832)	0.926		
**Pancreatic texture**		1.153 (0.175-4.425)	0.855		
**Tumor location (Ref: Pancreas)**			0.011		
	**Ampulla of Vater**	1.242 (0.173-6.024)	0.800		
	**Distal common bile duct**	4.720 (1.633-15.590)	0.006		
	**Duodenum**	NA*	0.989		
**Cancer**		1.777 (0.562-7.867)	0.377		
**Resection margin status**		3.905 (1.013-12.553)	0.030	4.821 (1.044-20.618)	0.034

* The results could not be calculated due to the existence of zero cells.
